# Study characteristical and regional influences on postpartum depression before vs. during the COVID-19 pandemic: A systematic review and meta-analysis

**DOI:** 10.3389/fpubh.2023.1102618

**Published:** 2023-02-15

**Authors:** Xiaoqian Zhang, Chun Wang, Xiaoli Zuo, Bert Aertgeerts, Frank Buntinx, Tang Li, Mieke Vermandere

**Affiliations:** ^1^Department of Public Health and Primary Care, Academic Center for General Practice, Katholieke Universiteit Leuven, Leuven, Belgium; ^2^Department of Family Medicine, Qingdao United Family Hospital, Qingdao, China; ^3^Department of Obstetrics and Gynecology, Qingdao Municipal Hospital, Qingdao, China; ^4^Department of Pediatrics, Qingdao University Medical College, Qingdao, China

**Keywords:** postpartum depression, depression, COVID-19, postpartum, influencing factors

## Abstract

**Background:**

While the public is under serious pressure from the coronavirus disease 2019 (COVID-19), the final impact and possible contributing factors to postpartum depression symptoms (PPDS) remain unknown. Therefore, a meta-analysis to investigate the association between PPDS and the COVID-19 pandemic was carried out by comparing the data between pre-pandemic and post-pandemic timeframes and exploring the influencing factors.

**Methods:**

This systematic review was prospectively registered and recorded in a study protocol (Prospero CRD42022336820, http://www.crd.york.ac.uk/PROSPERO). A comprehensive search of PubMed, Embase, Web of Science, CINALH, Cochrane and Scopus was cmpleted on June 6, 2022. Studies that compared the prevalence of PPD before and during the COVID-19 pandemic period were included.

**Results:**

Of 1766 citations identified, 22 studies were included with 15,098 participates before the COVID-19 pandemic and 11,836 participants during the COVID-19 pandemic. Overall, the analysis showed that the epidemic crisis was associated with an increased prevalence of PPDS (OR: 0.81 [0.68, 0.95], *P* = 0.009, *I*^2^ = 59%). Subgroup analysis was conducted according to the study characteristics and regions. Within the study characteristics classification, results showed an obvious increase in the prevalence of PPDS during the COVID-19 pandemic if PPDS cutoff was defined as Edinburgh postpartum depression score (EPDS) ≥13 points (OR: 0.72 [0.52, 0.98], *P* = 0.03, *I*^2^ = 67%) and an increased prevalence in follow-ups that happened after 2 weeks (≥ 2 weeks postpartum) (OR: 0.81 [0.68, 0.97], *P* = 0.02, *I*^2^ = 43%). Selected studies that were high-quality (OR: 0.79 [0.64, 0.97], *P* = 0.02, *I*^2^ = 56%) demonstrated an increased prevalence of PPDS during the COVID-19 pandemic period. Sorting by regional factors, studies conducted in Asia (OR: 0.81 [0.70, 0.93], *P* = 0.003, *I*^2^ = 0%) showed an increase of PPDS prevalence rates during the COVID-19 period, while studies conducted in Europe (OR: 0.82 [0.59, 1.13], *P* = 0.23, *I*^2^ = 71%) and North America (OR: 0.66 [0.42, 1.02], *P* = 0.06, *I*^2^ = 65%) showed no significant difference. All studies conducted in the developed (OR: 0.79 [0.64, 0.98], *P* = 0.03, *I*^2^ = 65%) and developing countries (OR: 0.81 [0.69, 0.94], *P* = 0.007, *I*^2^ = 0%) showed an increase of PPDS during the COVID-19 period.

**Conclusions:**

The COVID-19 pandemic is associated with an increased prevalence of PPDS, especially after long-term follow-up and among the group with a high possibility of depression. The negative influence from the pandemic, causing more PPDS was significant in studies from Asia.

## 1. Introduction

The Coronavirus disease 2019 (COVID-19) pandemic spread rapidly from Wuhan, China to the world and quickly developed into a global crisis (1). Confronted with the rapid increase in the number of infections and the uncertainty of the outcome, people in the whole world are facing varying degrees of psychological pressure. The fear of the COVID-19 infection and the strict isolation measures have produced a negative impact on the mental health of people (2, 3).

The term postpartum depression (PPD) refers to episodes of depression that usually appear within 4 weeks of delivery and can last for years (4). Postpartum women are more susceptible to clinical depression, marked by agitation, mood swings and sleep disorders (1, 5). Postpartum depression symptoms (PPDS) are one of the most common mental problems affecting 10–17% of postpartum individuals and causing significant morbidity and mortality (6–8). Suicide caused by postpartum mental problems accounts for about 20% of postpartum deaths (9). In addition, postpartum depression during this period can negatively influence the health and emotional development of newborns which can become a burden on both families and societies (10).

It is likely that those who already suffer from physical or mental health conditions, such as postpartum depression, will be most adversely affected by the current pandemic (11). Recently, numerous studies focused on the effect of the COVID-19 pandemic on PPDS, have produced conflicting results. Several studies demonstrated that the prevalence of PPDS is unchanged or even decreased during the COVID-19 pandemic (12–14), while others identified an increased prevalence (15–17). Yan et al. (18) included three studies in their meta-analysis and concluded that the prevalence of postpartum depression was 22%, while Shorey et al. (19) included five studies and demonstrated that the prevalence of postpartum depression was 17% during the COVID-19 period. Hessami et al. (20) evaluated the effect of the COVID-19 pandemic on the depression of women during pregnancy and the perinatal period through meta-analysis and found that the COVID-19 pandemic seems to have no significant influence on the diagnosis of depressive symptoms. Yet, only limited studies have compared the negative effect from the COVID-19 pandemic to postpartum women (21). Therefore, the goal of this systematic review and meta-analysis is to investigate the impact of the COVID-19 pandemic on the prevalence of PPDS by comparing the data from the pre-pandemic and post-pandemic timeframes, and exploring possible influencing factors from different subgroups of study characteristics and regions.

## 2. Methods

### 2.1. Protocol and registration

This systematic review was prospectively registered and recorded in a study protocol (Prospero CRD42022336820, http://www.crd.york.ac.uk/PROSPERO). This study was based on the Meta-Analysis of Observational Studies in Epidemiology (MOOSE) guidelines (22) and the Preferred Reporting Items for Systematic Reviews and Meta-Analyses (PRISMA) statement (23).

### 2.2. Eligibility criteria

Studies that reported the prevalence of PPDS during the postpartum period before and during the COVID-19 pandemic were included. Studies were eligible if they met the following criteria: (a) data were available during both pre- and post-pandemic timeframes and data for the postpartum period (b) utilized standardized and validated rating scales for measurement and (c) applied a clear cutoff for screening and defining PPDS. The time of the postpartum period was not limited. No restrictions were applied on the basis of the study setting, the study design, or the country.

Studies were excluded from this review if they met the following criteria: (a) not original articles such as commentaries, editorials, case reports, letters to editor, review studies, conference abstracts, etc.; (b) irrelevant studies or animal studies; (c) insufficient information was provided to calculate the prevalence of PPDS before and during the COVID-19 pandemic timeframe; (d) if a study included hypothetical or unquantified results (no supportive statistical evidence of analyzed outcome); (e) full-texts were inaccessible; or (f) data were presented in any language other than English.

### 2.3. Information sources

Six electronic databases (PubMed, Embase, Web of Science, CINALH, Cochrane and Scopus) were searched for related studies. Additional eligible articles were identified by manually searching cross-references of relevant articles (snowballing technique). The search was performed on June 6, 2022. We referred to the PICO-style approach and used combinations of the descriptors “postpartum/postnatal”, “depression/mental health” and “COVID-19 pandemic” in English, applying all their synonyms and associated word variants, and searching titles, abstracts, keywords, and the Mesh Terms. The search was limited to 2019 onwards, since COVID-19 officially started in 2019. A detailed approach of the search strategy is described in Appendix A.

### 2.4. Study selection

Search data were exported to a citation program (Endnote X9), duplicates were discarded, and the final Endnote file was uploaded to the internet intelligent systematic review tool (Rayyan). The cataloging and organizing of the evidence were performed separately by the two primary reviewers (XQ.Z, C.W). Disagreements were resolved by group discussion or consultation with a third party (M.V, B.A, F.B). The final assessment was carried out together by one primary and three senior authors (XQ.Z, M.V, B.A, F.B).

### 2.5. Data extraction

For previously published data, only the most comprehensive articles have been included in this meta-analysis. The extracted data was as follows: first author name, publication year, study country, study population, study type, study quality, study period, and population age (Table 1). Data extraction was performed by the two primary reviewers. Discrepancies were resolved by discussion and consensus with a third party. Authors of the original publications were contacted in cases of missing data.

**Table 1:** Characteristics of the included studies on postpartum depression (before COVID-19 vs. during COVID-19).

**References**	**Country**	**Study pop**	**Study type**	**NOS**	**Group**	**Study period**	**Sample size**	**Age**
Chrzan-Detkoś et al. (24)	Poland	≤1year	Pre-post	5	Pre	October 1–November 10, 2019	61	31.04 ± 3.70^*^
					During	February 20–March 30, 2020	78	31.74 ± 5.06^*^
Boekhorst et al. (25)	Netherlands	8–10 weeks	Pre-post	7	Pre	January 7, 2019–March 1, 2020	250	30.88 ± 3.67^*^
					During	March 1–May 14, 2020	59	30.75 ± 3.64^*^
Eberhard-Gran et al. (26)	Norway	2 weeks-13 months	Pre-post	6	Pre	November 2008 to April 2010	4,662	NR
					During	12 March 2020 to 12 April 2021	3,642	NR
Hiiragi et al. (27)	Japan	4-5 weeks	Pre-post	6	Pre	March 2019–June 2019	339	33 (29-37)^#^
					During	March 2020–June 2020	279	33 (29-37)^#^
Hui et al. (28)	China	Day 1 and 7	Pre-post	6	Pre	January 1, 2019– January 4, 2020	3,432	33.1 ± 4.4^*^
					During	January 5, 2019– April 30, 2020	925	33.1 ± 4.6^*^
Kuipers et al. (14)	Belgium	<52 weeks	Pre-post	7	Pre	August 8, 2019–February 3, 2020	456	30.53 ± 4.06^*^
					During	March 13, 2020–February 17, 2021	148	30.44 ± 3.7^*^
Layton et al. (29)	Canada	≤1 year	Pre-post	6	Pre	March, 2019– March, 2020	305	32.94 ± 5.17^*^
					During	April– October, 2020	298	31.74 ± 4.78^*^
Li et al. (30)	China	Week 6	Pre-post	7	Pre	October–December, 2019	546	29.36 ± 3.5^*^
					During	February–April, 2020	655	30.49 ± 3.7^*^
Loret de Mola et al. (31)	Brazil	11.4 ± 3.7^*^ months	Pre-post	5	Pre	January 1–December 31, 2019	1,136	NR
					During	May 11–July 20, 2020	1136	NR
Mariño-Narvaez et al. (32)	Spain	At most≤1 month	Pre-post	6	Pre	September 1, 2019–March 1, 2020	82	34.57 ± 4.81^*^
					During	April 1–July 1, 2020	75	33.84 ± 4.45^*^
Pariente et al. (13)	Israel	Day 2	Pre-post	6	Pre	October 2016–April 2017	123	28.3 ± 5.0^*^
					During	March 18–April 29, 2020	223	29.1 ± 5.1^*^
Perez et al. (17)	Germany	3–8 weeks	Pre-post	8	Pre	2016–2019	97	34.51 ± 3.25^*^
					During	March 15–May 1, 2020	65	36.02 ± 4.55^*^
Madera et al. (33)	Italy	Dhs	Pre-post	7	Pre	January, 2018–January, 2020	605	NR
					During	May, 2020–October, 2020	295	NR
Puertas-Gonzalez et al. (34)	Spain	≤1 month	Pre-post	7	Pre	March 2019–February 2020	96	32.96 ± 3.97^*^
					During	April 1–July 1, 2020	116	33.86 ± 4.60^*^
Sade et al. (35)	Israel	Dhs	Pre-post	7	Pre	November, 2016–April, 2017	84	NR
					During	March 19, 2020–May 26, 2020	279	NR
Sudhinaraset et al. (36)	Kenya	≤8 months	Pre-post	6	Pre	In 2019	1,014	NR
					During	After March 16, 2020	1,072	NR
Suzuki (12)	Japan	≤1 month	Pre-post	5	Pre	March 9–April 11, 2019	148	NR
					During	March 11–April 13, 2020	132	NR
Vatcheva et al. (37)	Belgium	3–6 months	Pre-post	6	Pre	January 1, 2017–December 31, 2019	108	30.0 ± 5.2^*^
					During	April 1, 2020–March 31, 2021	34	30.9 ± 5.1^*^
Waschmann et al. (38)	America	≤3 months	Pre-post	7	Pre	January 1–June 1, 2019	557	31.8 ± 5.33^*^
					During	January 1–June 1, 2020	504	31.4 ± 5.48^*^
Yakupova et al. (39)	Russia	≤14 months	Pre-post	7	Pre	January–February 2020	611	31.17 ± 4.54^*^
					During	February–March 2021	1,645	30.98 ± 4.42^*^
Zanardo et al. (15)	Italy	Day 2	Pre-post	7	Pre	March 8–May 3, 2019	101	32.98 ± 5.07^*^
					During	March 8–May 3, 2020	91	33.73 ± 5.01^*^
Zhang et al. (16)	Canada	6–10 weeks	Pre-post	8	Pre	March 1, 2019–February 29, 2020	285	33.9 ± 0.22^*^
					During	February 29, 2020–February 28, 2021	85	34.6 ± 0.45^*^

^*^Mean ± SD, ^#^median (interquartile range).

Study pop, Study population; Dhs, Duration of hospital stay; NOS, Newcastle-Ottawa scale; NR, not reported; Pre, Pre-COVID-19 pandemic; During: During-COVID-19 pandemic.

### 2.6. Risk of bias assessment

The two primary authors (XQ.Z/C.W) independently evaluated the risk of bias of the included studies using the Newcastle-Ottawa Scale (NOS) (40) to estimate the quality of the included studies. In the case of disagreement, a third party joined the discussion to determine a final decision. The final assessment was carried out together by three senior authors. Studies with a quality rating of five or greater were included in this meta-analysis. Studies with a score of seven or greater were classified as high-quality studies with the others being classified as moderate-quality studies (Table 1).

### 2.7. Statistical analysis and synthesis of results

The statistical software programs, Review Manager (RevMan 5.3) and Stata 12.0, were used for statistical analysis in this study. Chi-squared statistics and *I*^2^ tests were used to calculate heterogeneity (41) and the random effects model was used to deal with anticipated clinical heterogeneity. We conducted a sensitivity analysis to evaluate the effect of each included study by the comman of “metaninf” on Stata 12.0 (Supplementary Image 1). The Egger's test (42) and the Begg's test (43) were used to evaluate potential publication bias.

### 2.8. Subgroup analyses

Two predefined subgroup analyses and four exploratory subgroup analysis requested by the two primary reviewers (XQ.Z, C.W) were carried out based on the following classifications:

Study characteristics:

A. Cut-off value of different assessment scales [predefined; cut-off value 9-11 and cut-off value ≥13 of Edinburgh Postnatal Depression Scale (EPDS)].
B. Follow-up duration (predefined; within 2 weeks after delivery and more than 2 weeks after delivery).
C. Different study quality (exploratory; high-quality studies and moderate-quality studies).

Regional subgroups

D. Study continents (exploratory; Europe vs. Asia vs. North America).
E. Different country types (exploratory; developed countries vs. developing countries).

## 3. Results

### 3.1. Description of studies

#### 3.1.1. Study selection

**Figure 1:**
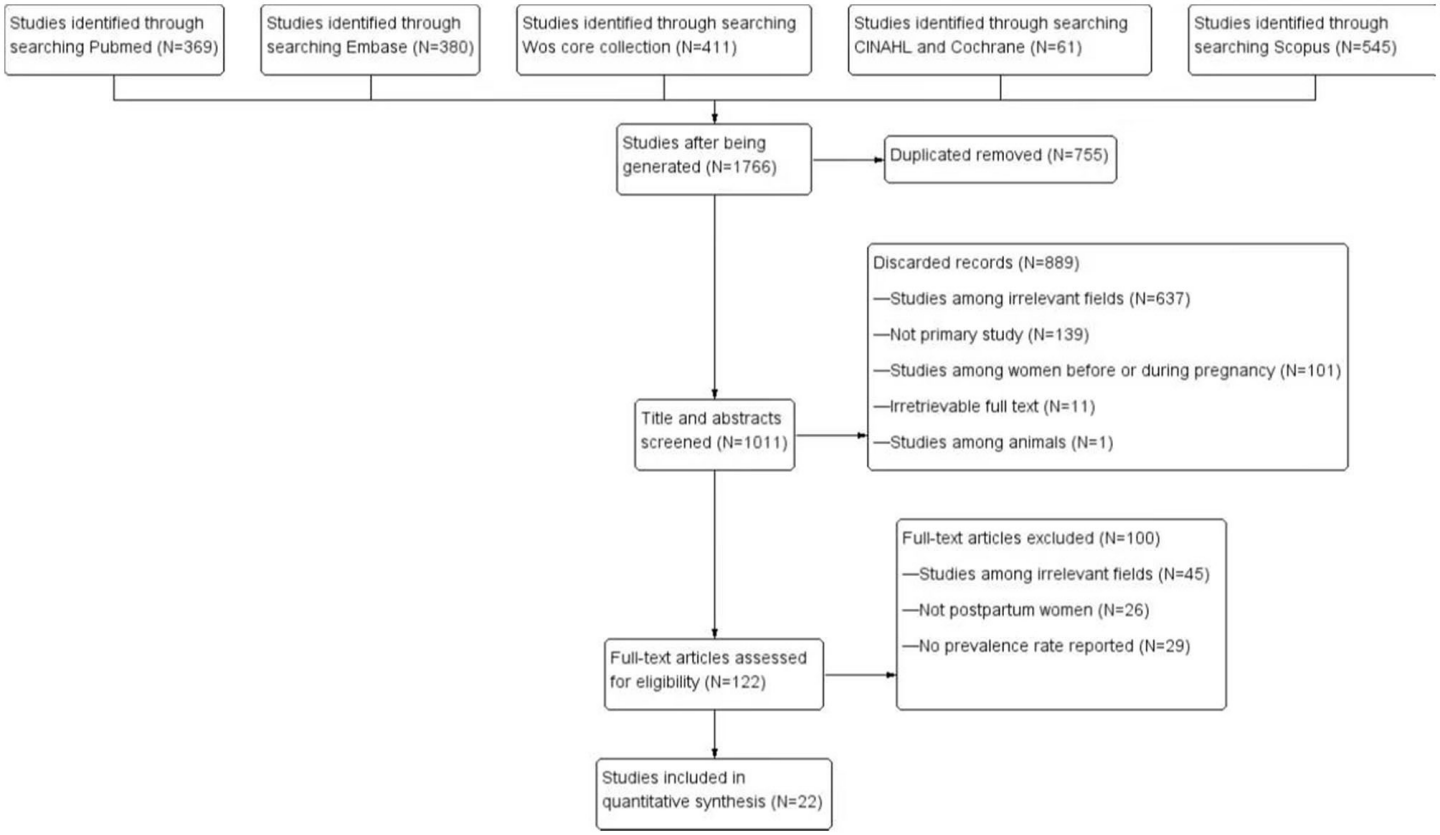
PRISMA study selection flow diagram.

The initial search provided a total of 1,766 articles of which 755 were deleted because of duplication. Another 1,011 articles were then excluded after screening titles and abstracts, and 122 items were evaluated for eligibility. Ultimately, 22 (12–17, 24–32, 34–39, 44) studies were selected for quantitative synthesis. Figure 1 demonstrated the detailed process of the literature search. The follow-up duration ranged from one day after delivery to 13 months after delivery in different studies.

#### 3.1.2. Study characteristics

Table 1 outlines the detail characteristics of the included studies. A total of 22 studies with 11,836 participants during the COVID-19 pandemic timeframe and 15,098 before the COVID-19 pandemic timeframe were included in this meta-analysis. Among the 22 studies, 11 studies were conducted in Europe [two in Belgium (14, 37), two in Italy (15, 33), two in Spain (32, 34), one in Russia (39) and remaining four were in Germany (17), Norway (26), Poland (24) and Netherlands (25)], six studies were conducted in Asia [two in Japan (12, 27), two in China (28, 30) and two in Israel (13, 35)], three studies were conducted in North America [two in Canada (16, 29) and one in the United States (38)] and the remaining two studies were conducted in Brazil (31) and Kenya (36). Four of the included studies were conducted in developing countries (28, 30, 31, 36) and the remaining 18 studies were conducted in developed countries. Five of the included studies detected the mental health of women within 2 weeks postpartum (13, 15, 28, 33, 35) and the other 17 studies recorded the mental health of the women within a longer time after delivery. The study quality of 11 studies were high (14–17, 25, 30, 32, 33, 35, 38, 39) and the other 11 studies were moderate. Nine studies which used the EPDS scale chose a cut-off value of 9–11 (12, 17, 27, 28, 32, 34, 35, 38, 39), while another eight studies chose a cut-off value of ≥13 (14–16, 25, 29–31, 33). Three studies compared different cut-off values (9–11 and ≥13) (13, 24, 37). Among the 22 studies, 20 studies assessed PPDS by using the standard EPDS scale, while one study (36) assessed postpartum depression by using the World Health Organization's Maternal Tool and the other one used a short form of EPDS scale (EPDS-4) (26). The data of three studies were not quantitatively analyzed due to the fact that two study (26, 36) used a different measuring system, while the other study (37) only looked at mothers of extreme and early preterm infants. At this moment, 19 studies were included in the further meta-analysis (8, 12–17, 24, 25, 27–33, 35, 38, 39).

### 3.2. Risk of bias in included studies

All studies included in the meta-analysis used EPDS as a measuring scale and compared information from pre-pandemic and post-pandemic timeframes. The qualities of the eligible studies were evaluated based on the Newcastle-Ottawa Scale (NOS) assessment tool and studies with a quality assessment score above four (low quality = 0–3; moderate quality = 4–6; high quality = 7–9) were included in the review. Table 1 shows a summary of the quality assessment of all studies included in the meta-analysis using the NOS tool.

### 3.3. Data synthesis and statistical analysis

#### 3.3.1. Overall analysis

**Figure 2:**
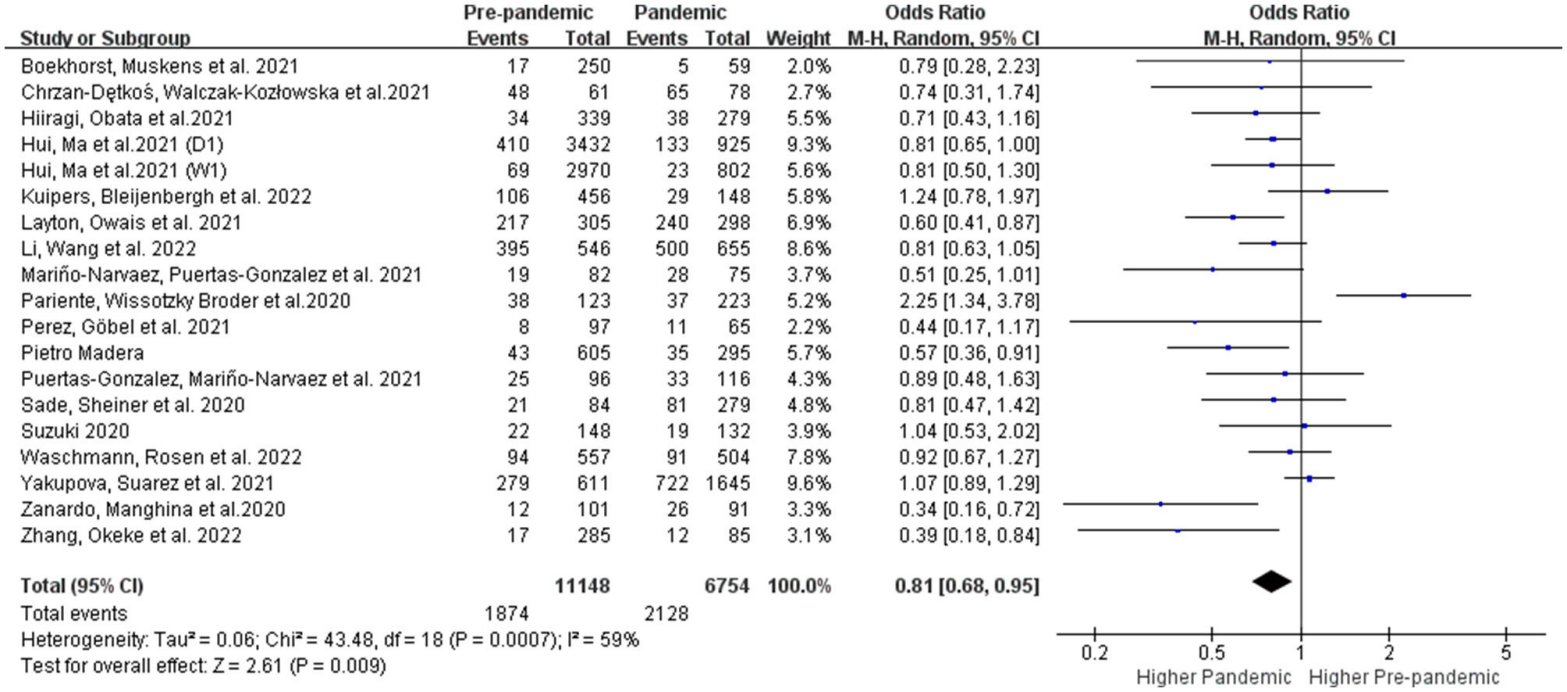
Overall meta-analysis comparing the prevalence of PPDS before and during the COVID-19 pandemic.

Nineteen of the twenty-two selected studies reported PPDS using the standard EPDS scale during the pre-pandemic and post-pandemic timeframes. Furthermore, we conducted a sensitivity analysis and deleted one study (31) because of an apparent deviation (Supplementary Image 1). In the end, only 18 studies were included in the final meta-analysis (12–17, 24, 25, 27–33, 35, 38, 39). Overall, the epidemic crisis is associated with an increased prevalence of PPDS (OR: 0.81 [0.68, 0.95], *P* = 0.009) though the high heterogeneity in the forest plot (I^2^ = 53%) (Figure 2). We used Stata 12.0 software to examine the publication bias using Begg's funnel plots and no publication bias was discovered (Begg's test: *P* = 0.130; Egger's test: *P* = 0.325).

#### 3.3.2. Subgroup analysis

##### 3.3.2.1. Subgroup analysis in study characteristics

###### 3.3.2.1.1. Subgroups with different cut-offs

**Figure 3:**
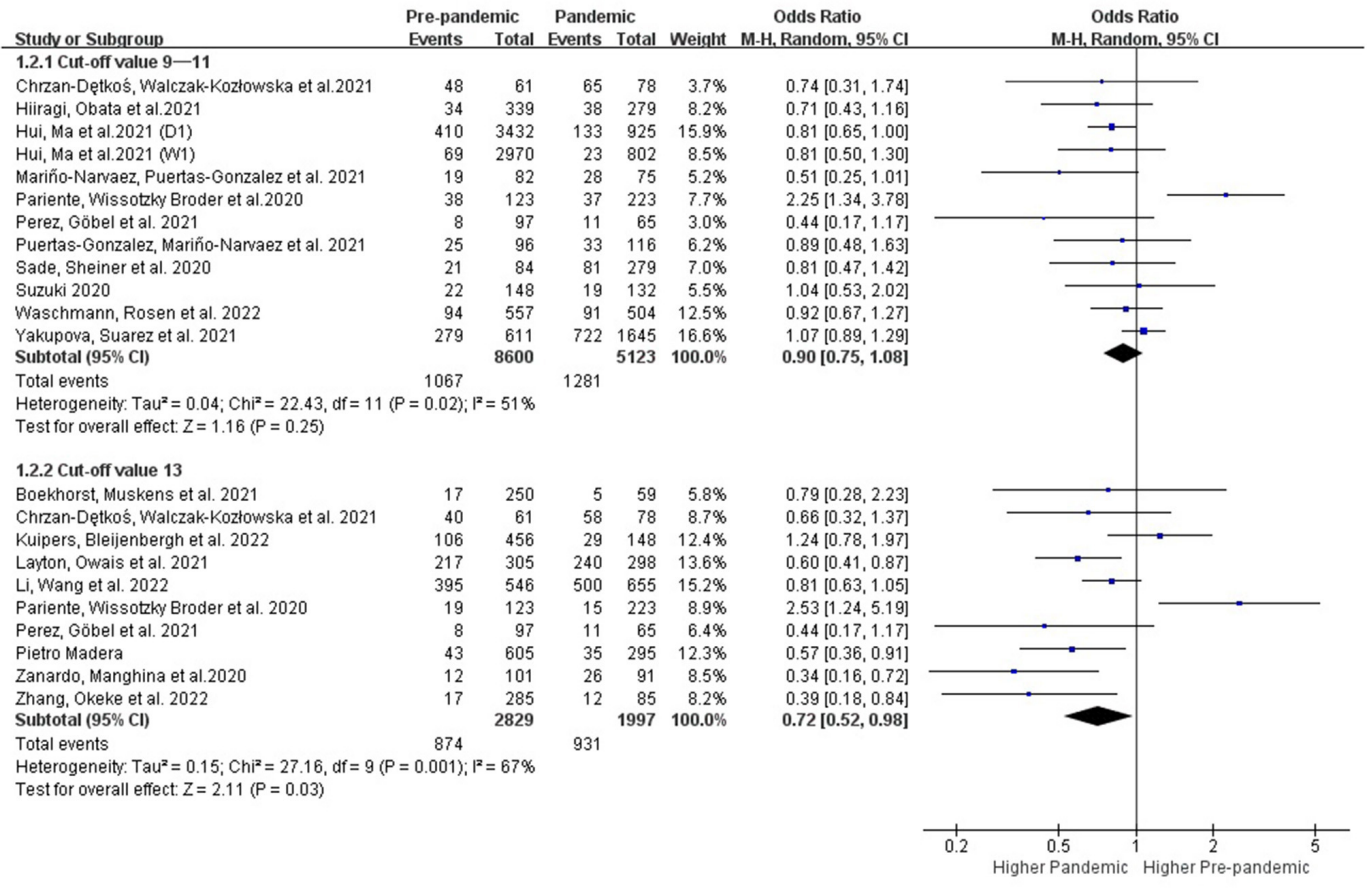
Subgroup analysis comparing the prevalence of PPDS before and during the COVID-19 pandemic according to the cut-off values of EPDS.

Two cut-off points for EPDS were commonly used in the literature: ≥10 points was more suitable and sensitive for routine use in a primary setting for detecting PPDS, whereas ≥13 indicated that a likely case of PPDS of varying severity required an extended clinical examination (45). We conducted the subgroup analysis according to the different cut-off values of EPDS. As shown in Figure 3, women delivering during the COVID-19 pandemic had no significant increase in the prevalence of PPDS with the cut-off value of EPDS at 9–11 (OR: 0.90 [0.75, 1.08], *P* = 0.25, *I*^2^ = 51%) compared to women delivering before the pandemic. However, the prevalence of PPDS defined as EPDS≥13 points (OR: 0.72 [0.52, 0.98], *P* = 0.03, *I*^2^ = 67%) showed an obvious increase during the COVID-19 pandemic (Figure 3).

###### 3.3.2.1.2. Subgroups with different follow-up times

**Figure 4:**
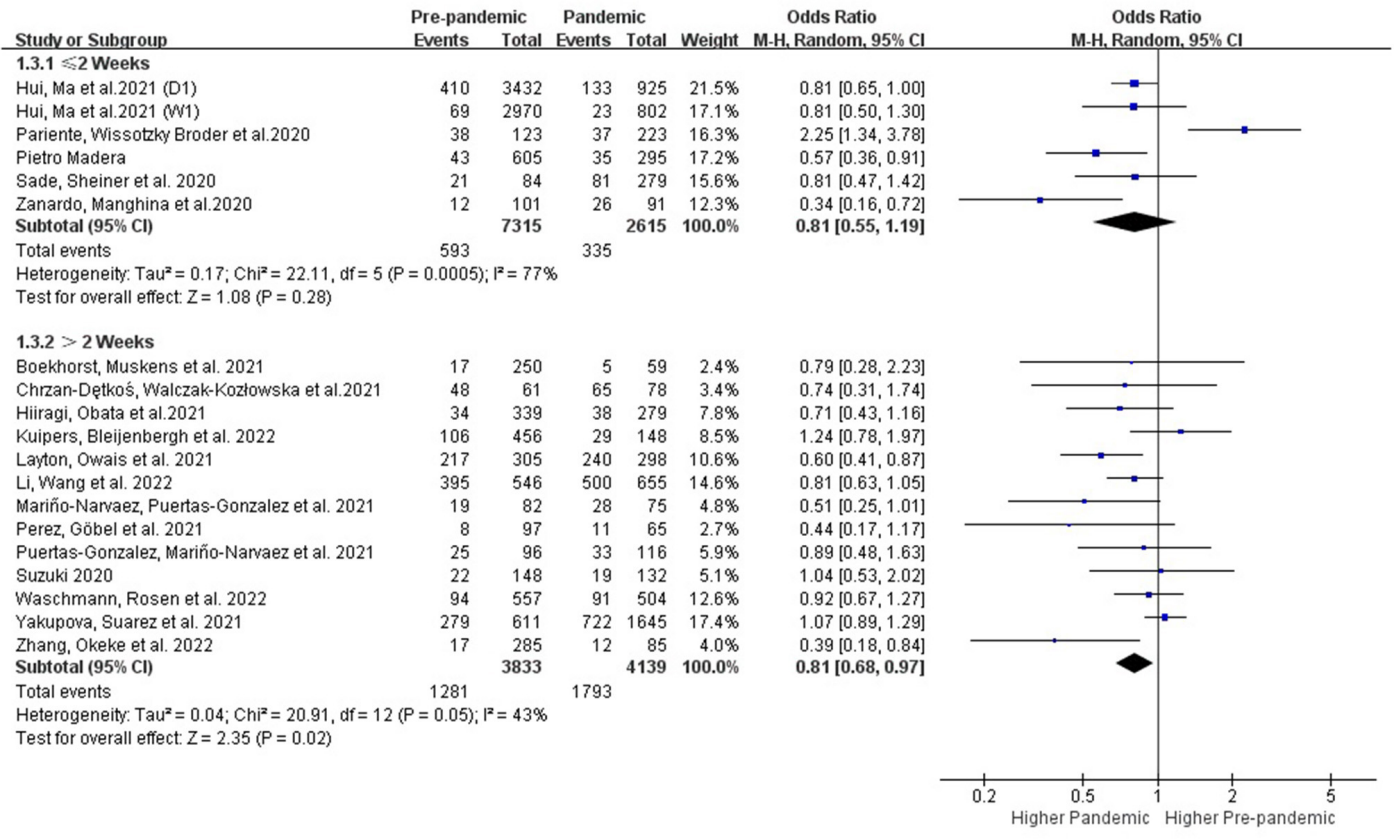
Subgroup analysis comparing the prevalence of PPDS before and during the COVID-19 pandemic according to postpartum time.

Postpartum depression will generally manifest within 2 weeks immediately after delivery with a peak incidence on the fifth day, and it can last year's (46, 47). Further subgroup analysis with different follow-up times was performed. Results showed that the prevalence of PPDS within 2 weeks of delivery showed no change before and during the COVID-19 period (OR: 0.81 [0.55, 1.19], P = 0.28, *I*^2^ = 77%), while the prevalence of postpartum depression showed an obvious increase when detected after the first 2 weeks during the COVID-19 period (OR: 0.81 [0.68, 0.97], P = 0.02, *I*^2^ = 43%) (Figure 4).

###### 3.3.2.1.3. Subgroups with different study quality

**Figure 5:**
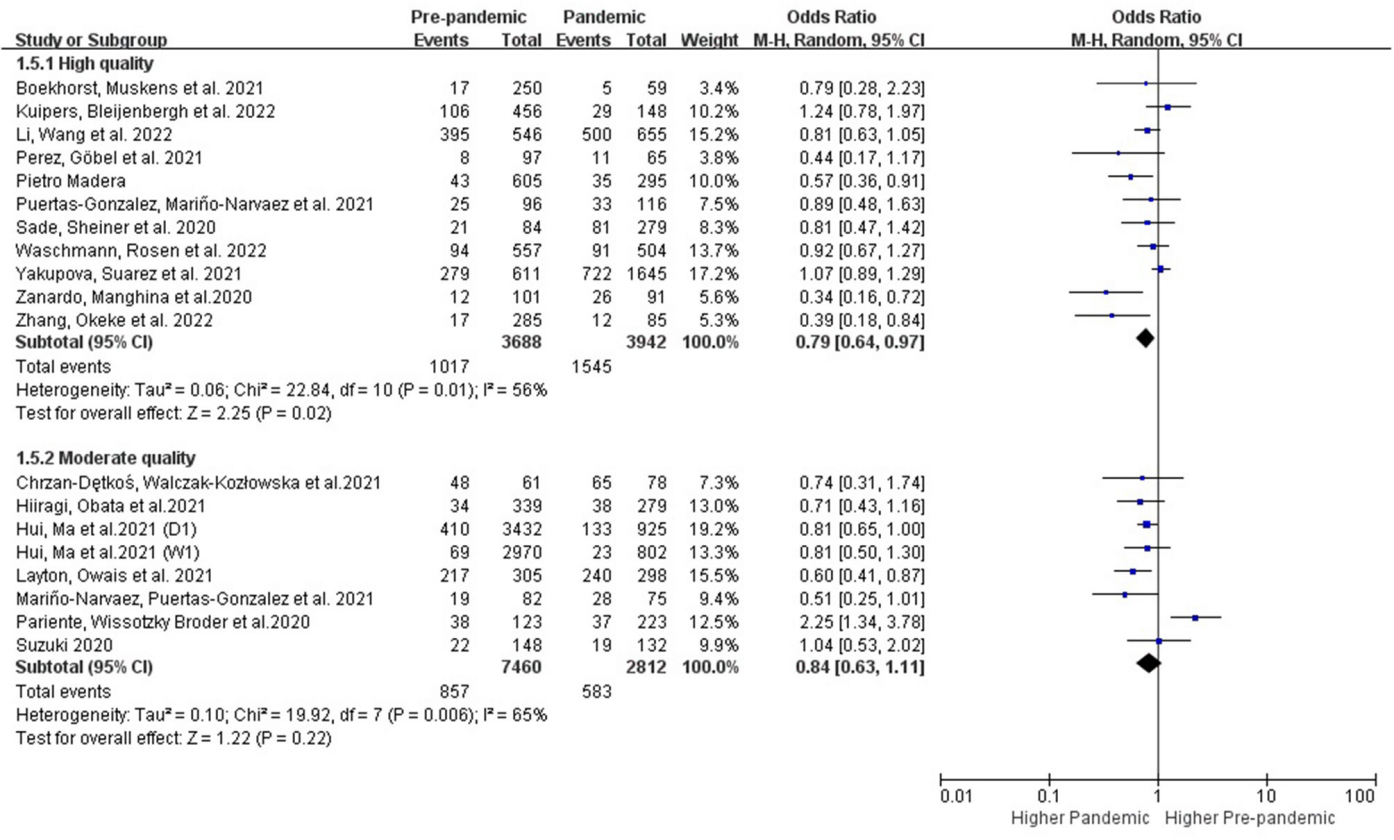
Subgroup analysis comparing the prevalence of PPDS before and during the COVID-19 pandemic according to study quality.

Statistical heterogeneity among different subgroups based on the study quality was evaluated by *I*^2^ of Higgins (Figure 5). The results of the studies that were high-quality (OR: 0.79 [0.64, 0.97], *P* = 0.02, *I*^2^ = 56%) demonstrated an increased prevalence of PPDS during the COVID-19 pandemic period, while the prevalence of PPDS showed no significant increase in those studies with moderate quality.

##### 3.3.2.2. Subgroup analysis in regions

###### 3.3.2.2.1. Subgroups with different regions and country types

**Figure 6:**
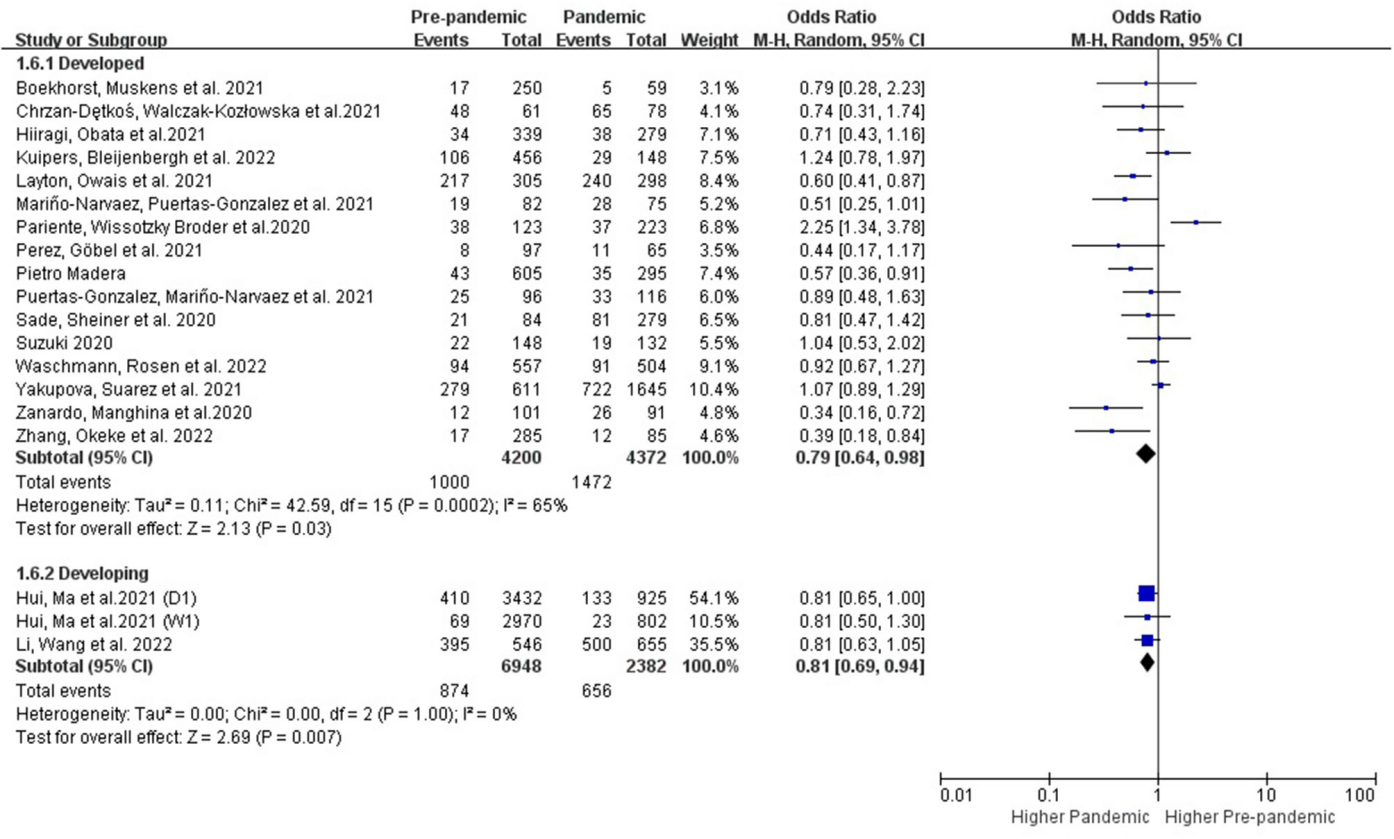
Subgroup analysis comparing the prevalence of PPDS before and during the COVID-19 pandemic according to different country types.

**Figure 7:**
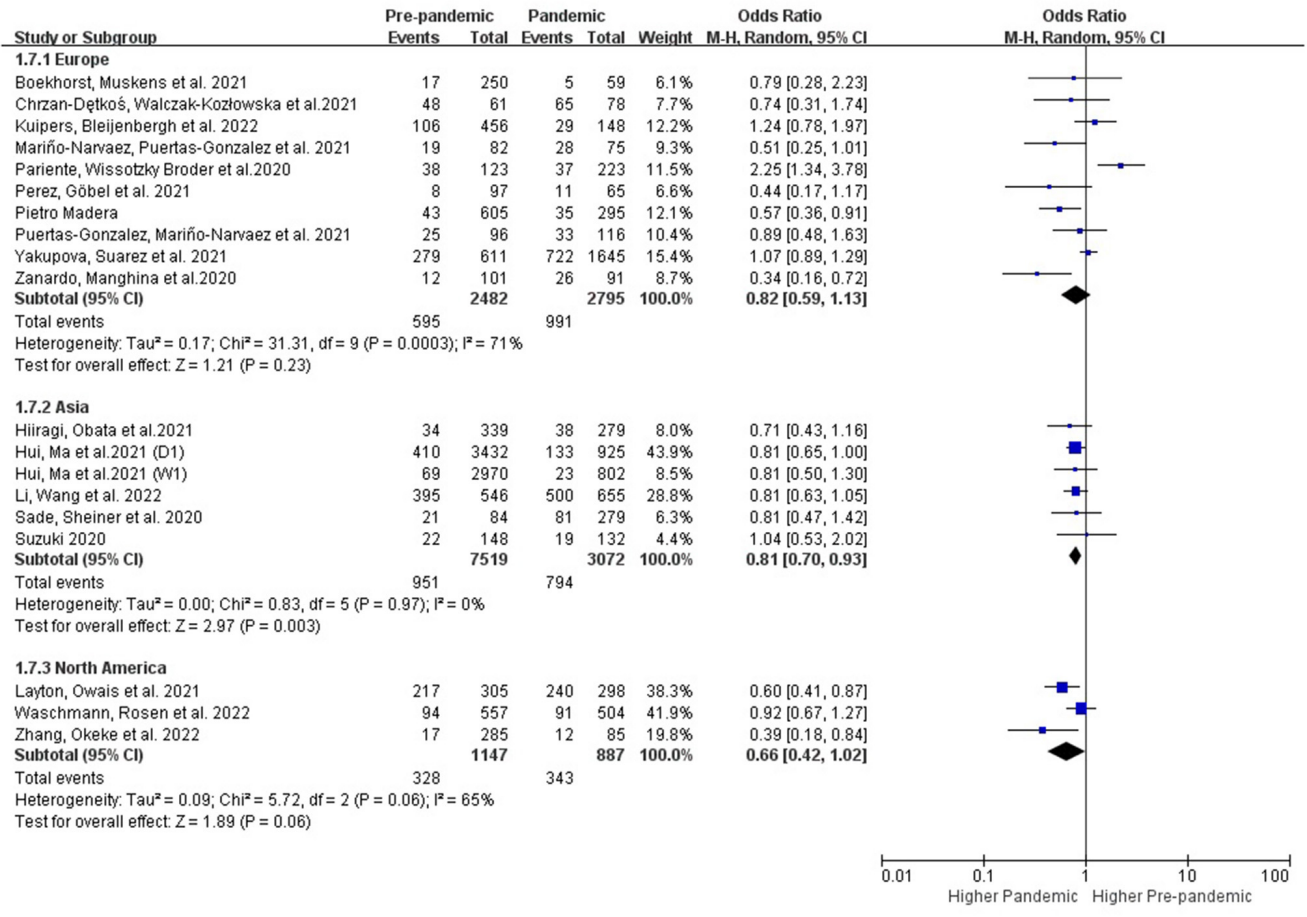
Subgroup analysis comparing the prevalence of PPDS before and during the COVID-19 pandemic according to different continents.

The studies conducted in the developed (OR: 0.79 [0.64, 0.98], *P* = 0.03, *I*^2^ = 65%) and developing countries (OR: 0.81 [0.69, 0.94], *P* = 0.007, *I*^2^ = 0%) showed an increase of PPDS during the COVID-19 period (Figure 6). Studies conducted in Asia (OR: 0.81 [0.70, 0.93], *P* = 0.003, *I*^2^ = 0%) showed an increase of PPDS during the COVID-19 period, while studies conducted in Europe (OR: 0.82 [0.59, 1.13], P = 0.23, *I*^2^ = 71%), and North America (OR: 0.66 [0.42, 1.02], P = 0.06, *I*^2^ = 65%) showed no significant difference. Further evidence is needed to examine potential differences in these subgroups (Figure 7).

## 4. Discussion

This meta-analysis compared the prevalence of PPDS by using data collected from both pre-pandemic and post-pandemic COVID-19 timeframes and explored the possible influence of study characteristics and regions. Results demonstrated that the COVID-19 pandemic is associated with an increased prevalence of PPDS, especially in the studies where a high risk of PPDS was defined as EPDS ≥ 13 points and detected >2 weeks after delivery. Meanwhile, Studies that were high-quality and conducted in Asia showed an increase of PPDS during the COVID-19.

### 4.1. Subgroups with different cut-offs

Diagnosis of postpartum depression requires a clinical interview where EPDS is used to indicate postpartum depressive symptoms. Different cut-offs were used with varying sensitivity and specificity (45). The results of the meta-analysis showed that a cut-off value of 10/11 had the greatest sensitivity and specificity for assessing the risk of a mild depressive disorder in perinatal women (48). A score of 9 or above is often used to indicate the likelihood of postpartum depression in Japanese women (49). In this study, we conducted a further subgroup analysis evaluating the different cut-off values of EPDS (Figure 3). Results demonstrated that women delivering during the COVID-19 pandemic had no significant increase in the prevalence of PPDS with the cut-off value of 9–11 compared to women delivering before the pandemic. However, the prevalence of PPDS defined as EPDS ≥13 points had an obvious increase during the COVID-19 pandemic, suggesting that postpartum women who are more likely to suffer from a depressive illness might be more susceptible to the negative impacts of the COVID-19 pandemic.

### 4.2. Subgroups with different follow-up times

Postpartum depression, by definition, starts within 4 weeks postpartum and symptoms must persisting for at least 2 weeks (50). However, depressive symptoms that start beyond 4 weeks after delivery can still cause harm and require treatment. Assessment for postpartum depression is still recommended, in clinical practice, within the first 12 months after delivery (51). About 70% of new mothers experience mild depressive symptoms postpartum which usually begins to subside spontaneously within 2 weeks without significant impairment of function or continued psychotic symptoms (46). In this study, we conducted further subgroup analysis according to the postpartum time, and results showed that the prevalence of PPDS within 2 weeks after delivery showed no difference before and during the COVID-19 period, while the prevalence of postpartum depression increased obvious during the COVID-19 pandemic period when detected 2 weeks after delivery (Figure 4). Based on the current research results, the impact of the COVID-19 pandemic on PPDS is more long-lasting and lactating women should be provided with adequate psychological support to reduce the long-term negative effects of this pandemic.

### 4.3. Subgroups with different study quality

We also evaluated the heterogeneity based on the study quality. Subgroup analysis of these studies with high quality provided further evidence of the negative impact of the pandemic on PPDS, while no significant effect was shown in studies with moderate quality.

### 4.4. Subgroups with different regions

Our study found an increase of PPDS during the COVID-19 pandemic period in Asia (*I*^2^ = 0%), while studies conducted in Europe and North America showed no significant increase of PPDS prevalence (Figure 7). One recent meta-analysis that found an increased PPDS prevalence during the COVID-19 over the world with high heterogeneity (*I*^2^ = 95%) (21). No further subgroups analysis were conducted in this meta-analysis (21). Furthermore, the high heterogeneity between studies that may negatively affect the accuracy of the result. The robustness of the results from this meta-analysis was controlled by conducting sensitivity analysis. Studies that utilized different screening tools and studies that focused only on mothers of extreme and early preterm infants were excluded to achieve better analysis results (26, 36, 37).

### 4.5. Subgroups with different country types

Both of the study subgroups that were conducted in developing and developed countries showed a significant negative impact from the COVID-19 to the prevalence of postpartum depression (Figure 6). The number of studies in developing subgroups was limited, further evidence is needed to examine potential differences in these subgroups.

In addition to the factors mentioned above, strict isolation measures, level of education, economy, and social support, etc. can also have a negative impact on the mental health of the postpartum women (52, 53). Due to the limitations of the included studies, we were unable to further analyze these possible influencing factors. Multi-center high-quality research with large-sample sizes and perhaps, longitudinal research methods should be considered for future studies.

## 5. Strengths and limitations

This systematic review and meta-analysis compared the prevalence of PPDS before versus during the COVID-19 pandemic and explored possible influencing factors on postpartum depression from study characteristics and regions. Statistical analysis was carried out within different subgroups to demonstrate the possible negative effects from the COVID-19 pandemic on PPDS. However, it is undeniable that this research has some limitations. Firstly, many of the included studies collected their relevant data using internet surveys due to the quarantine policies during the pandemic which may have given rise to selection and decision biases. Secondly, some of the included studies did not depict the severity of the epidemic situation and the isolation measures during the study period which are all important influencing factors to mental health. Lastly, the limited number and the high heterogeneity of included articles also potentially affected the accuracy of the results.

## 6. Conclusion

This meta-analysis compared the prevalence of PPDS before vs. during the COVID-19 pandemic. Subgroup analyses were conducted to further examine the possible influencing factors. The results of subgroup analysis showed that the COVID-19 pandemic was associated with an increased prevalence of PPDS, especially in long-term follow-up studies (>2 weeks), in studies with high possibility of PPDS (EPDS ≥ 13) and in studies from Asia. Therefore, policymakers and health planners should give high priority to the mental health of this vulnerable group during a global health crisis and develop better support and assurance measures to deal with the impact of a pandemic on postpartum women.

## Author contributions

XZh, CW, BA, FB, and MV designed the review. XZh and CW contributed to conception and design of the study, data acquisition, and analysis and interpretation of data. XZh participated in writing the first draft of the paper. TL, BA, and FB revised critically for important intellectual content and gave final approval of the version to be published. All authors contributed to the intellectual content, read, and approved the final manuscript.

## Supplementary material

Supplementary Image 1 Meta-influence analyses of the included studies before **(A)** and after **(B)** the apparently deviated study was deleted.
