# Supplementary figures and images for: Study characteristical and regional influences on postpartum depression before vs. during the COVID-19 pandemic: A systematic review and meta-analysis
Source: Front Public Health. 2023 Feb 15;11:1102618. doi: 10.3389/fpubh.2023.1102618 (PMC9975262; doi:10.3389/fpubh.2023.1102618)

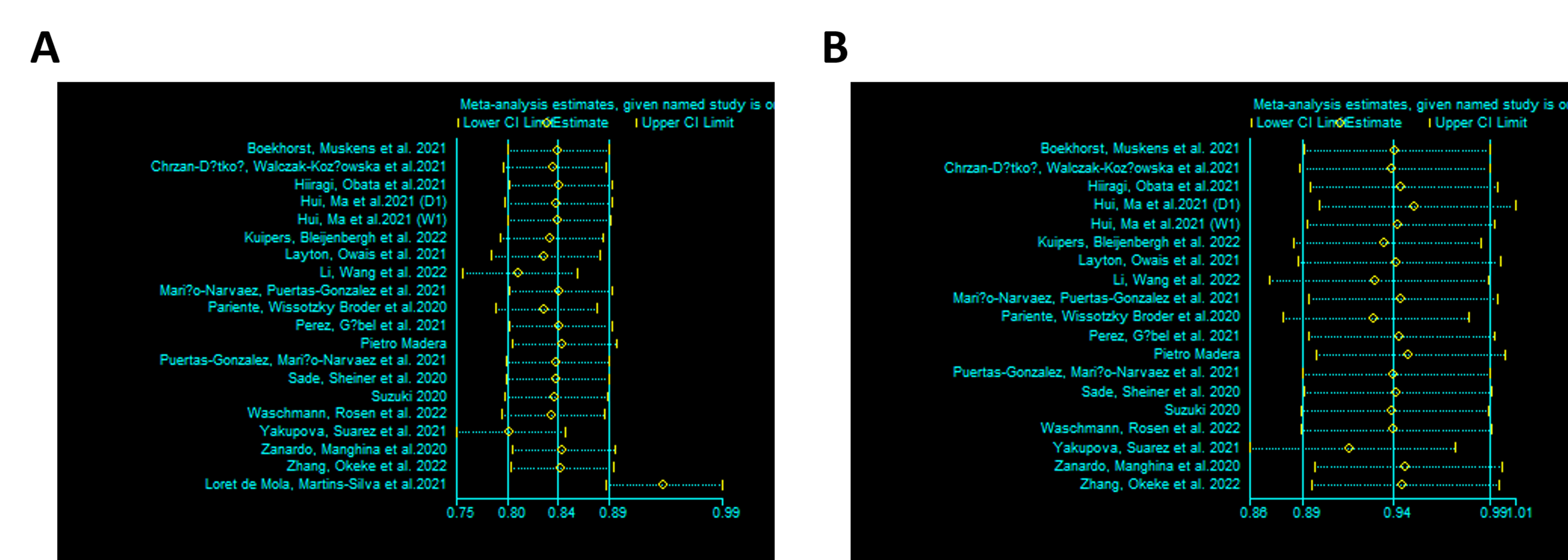

Supplement: Supplementary Image 1 — Meta-influence analyses of the included studies before (A) and after (B) the apparently deviated study was deleted. [file Image_1.TIF]
