# Supplementary material for: Study characteristical and regional influences on postpartum depression before vs. during the COVID-19 pandemic: A systematic review and meta-analysis
Source: Front Public Health. 2023 Feb 15;11:1102618. doi: 10.3389/fpubh.2023.1102618 (PMC9975262; doi:10.3389/fpubh.2023.1102618)
Supplement: Supplementary file 2 [file Data_Sheet_1.PDF]

Search Strings:

PubMed (including MEDLINE)

Concept 1

("Postpartum Period"[Mesh:NoExp] OR "Postpartum"[tiab] OR "Post-partum"[tiab] OR "Post-Natal"[tiab] OR "Postnatal"[tiab] OR "Puerper*"[tiab] OR "after birth"[tiab] OR "after delivery"[tiab])

Concept 2

(("COVID-19"[MeSH] OR nCoV[tiab] OR 2019nCoV[tiab] OR COVID[tiab] OR COVID19[tiab] OR SARS2[tiab] OR "cov 2"[tiab] OR cov2[tiab] OR coronavirus*[tiab] OR "corona virus*"[tiab] OR "severe acute respiratory syndrome 2"[tiab] OR "Wuhan virus"[tiab] OR nCoV[ad] OR 2019nCoV[ad] OR COVID[ad] OR COVID19[ad] OR "SARS-Cov-2"[MeSH] OR SARS2[ad] OR "severe acute respiratory syndrome 2"[ad] OR "cov 2"[ad] OR cov2[ad] OR coronavirus*[ad] OR "corona virus*"[ad] OR ((wuhan[tiab] OR novel[tiab] OR new[tiab] OR 19[tiab] OR 2019[tiab] OR epidem*[tiab] OR pandem*[tiab] OR outbreak[tiab] OR wuhan[ad] OR novel[ad] OR new[ad] OR 19[ad] OR 2019[ad] OR epidemy[ad] OR epidemic*[ad] OR pandem*[ad] OR outbreak[ad]) AND ("Coronavirus"[Mesh:NoExp] OR "Betacoronavirus"[Mesh:NoExp] OR "Coronavirus Infections"[Mesh:NoExp] OR "pneumonia virus*"[tiab] OR cov[tiab] OR hcov[tiab])))

AND 2019/12[PDAT]:2030[PDAT])

Concept 3: Depression

("Depressive Disorder"[Mesh:NoExp] OR "Depression"[Mesh] OR "Psychology"[Mesh] OR "psychology" [Subheading] OR "Mental Health"[Mesh] OR Psycholog*[tiab] OR "psychosocial"[tiab] OR "Mental"[tiab] OR Depressi*[tiab] OR "social inability"[tiab] OR "social ability"[tiab] OR "emotional"[tiab] OR "affective"[tiab])

Concept 4: Postpartum depression

"Depression, Postpartum"[Mesh]

((1 AND 3) OR 4) AND 2

Embase

Concept 1

('puerperium'/exp OR 'puerperium':ti,ab,kw OR ‘postpartum period’:ti,ab,kw OR ‘postpartum’:ti,ab,kw OR ‘post-partum’:ti,ab,kw OR ‘post-natal’:ti,ab,kw OR ‘postnatal’:ti,ab,kw OR ‘puerper*’:ti,ab,kw OR ‘after birth’:ti,ab,kw OR ‘after delivery’:ti,ab,kw)

Concept 2

('coronavirus disease 2019'/exp OR nCoV:ti,ab,kw,ad,ff OR 2019nCoV:ti,ab,kw,ad,ff OR COVID:ti,ab,kw,ad,ff OR COVID19:ti,ab,kw,ad,ff OR 'Severe acute respiratory syndrome coronavirus 2'/exp OR 'severe acute respiratory syndrome 2':ti,ab,kw,ff OR 'sars cov 2':ti,ab,kw,ad,ff OR SARS2:ti,ab,kw,ad,ff OR 'cov 2':ti,ab,kw,ad,ff OR cov2:ti,ab,kw,ad,ff OR coronavirus*:ti,ab,kw,ad,ff OR ‘corona virus*’:ti,ab,kw,ad,ff OR ‘wuhan virus’:ti,ab,kw OR ((wuhan:ti,ab,kw,ad,ff OR novel:ti,ab,kw,ad,ff OR 19:ti,ab,kw,ad,ff OR 2019:ti,ab,kw,ad,ff OR epidem*:ti,ab,kw OR epidemy:ti,ab,kw,ff OR epidemic*:ti,ab,kw,ad,ff OR pandem*:ti,ab,kw,ad,ff OR outbreak:ti,ab,kw,ad,ff OR new:ti,ab,kw,ad,ff) AND ('pneumonia virus*':ti,ab,kw OR cov:ti,ab,kw OR hcov:ti,ab,kw)))

AND [2019-2021]/py

Concept 3

('depression'/exp OR 'mental health'/exp OR 'psychology'/exp OR 'dysthymia':ti,ab,kw OR 'melancholia':ti,ab,kw OR 'depressi*':ti,ab,kw OR 'affective':ti,ab,kw OR 'mental':ti,ab,kw OR 'personality disorder':ti,ab,kw OR 'psychosocial':ti,ab,kw OR 'emotional':ti,ab,kw OR 'social inability':ti,ab,kw OR 'psychic':ti,ab,kw)

Concept 4

'postnatal depression'/exp OR 'maternal depression':ti,ab,kw OR 'post partum depression':ti,ab,kw OR ‘post-natal depression’:ti,ab,kw OR ‘postpartum depression’:ti,ab,kw OR ‘puerperal depression’:ti,ab,kw OR ‘puerperium depression’:ti,ab,kw

((1 AND 3) OR 4) AND 2

WOS core collection

Concept 1

TS=("Postpartum" OR "Post-partum" OR "Post-Natal" OR "Postnatal" OR "Puerper*" OR "after birth" OR "after delivery")

Concept 2

(TS=("nCoV" OR "2019nCoV" OR "COVID" OR "COVID19" OR "SARS2" OR "cov 2" OR "cov2" OR "coronavirus*" OR "corona virus*" OR "severe acute respiratory syndrome 2" OR "wuhan virus" OR (("wuhan" OR "novel" OR "19" OR "2019" OR "epidem*" OR "pandem*" OR "outbreak" OR "new") AND ("pneumonia virus*" OR "cov" OR "hcov"))) OR AD=("nCoV" OR "2019nCoV" OR "COVID" OR "COVID19" OR "SARS2" OR "cov 2" OR "cov2" OR "coronavirus*" OR "corona virus*" OR "severe acute respiratory syndrome 2") OR

(AD=("wuhan" OR "novel" OR "new" OR "19" OR "2019" OR "epidemy" OR "epidemic*" OR "pandem*" OR "outbreak") AND TS=("pneumonia virus*" OR "cov" OR "hcov")))

AND PY=(2019-2022)

Concept 3

TS=(Psycholog* OR "psychosocial" OR "Mental" OR Depressi* OR "social inability" OR "social ability" OR "emotional" OR "affective")

Concept 4

TS=("maternal depression" OR "post partum depression" OR "post-natal depression" OR "postpartum depression" OR "puerperal depression" OR "puerperium depression")

((1 AND 3) OR 4) AND 2

Scopus

Concept 1

TITLE-ABS-KEY ((postpartum) OR (post-partum) OR (post-natal) OR (postnatal) OR (puerper*) OR (after AND birth) OR (after AND delivery))

Concept 2

TITLE-ABS-KEY ((nCoV) OR (2019nCoV) OR (COVID) OR (COVID19) OR (SARS2) OR (cov 2) OR (cov2) OR (coronavirus*) OR (corona virus*) OR (severe acute respiratory syndrome 2) OR (wuhan virus) OR (((wuhan) OR (novel) OR (19) OR (2019) OR (epidem*) OR (pandem*) OR (outbreak) OR (new)) AND ((pneumonia virus*) OR (cov) OR (hcov)))) OR Affil ((Wuhan) OR (19) OR (novel) OR (new) OR (2019) OR (epidemy) OR (epidemic*) OR (pandem*) OR (outbreak)) AND TITLE-ABS-KEY ((pneumonia virus*) OR (COV) OR (hcov))

Concept 3

TITLE-ABS-KEY ((Psycholog*) OR (psychosocial) OR (Mental) OR (Depressi*) OR (social inability) OR (social ability) OR (emotional) OR (affective))

Concept 4

TITLE-ABS-KEY ((maternal depression) OR (post partum depression) OR (post-natal depression) OR (postpartum depression) OR (puerperal depression) OR (puerperium depression))

((1 AND 3) OR 4) AND 2

(((TITLE-ABS-KEY ((postpartum) OR (post-partum) OR (post-natal) OR (postnatal) OR (puerper*) OR (after AND birth) OR (after AND delivery))) AND (TITLE-ABS-KEY ((psycholog*) OR (psychosocial) OR (mental) OR (depressi*) OR (social AND in ability) OR (social AND ability) OR (emotional) OR (affective)))) OR (TITLE-ABS-KEY ((maternal AND depression) OR (post AND partum AND depression) OR (post-natal AND depression) OR (postpartum AND depression) OR (puerperal AND depressi on) OR (puerperium AND depression)))) AND (TITLE-ABS-KEY ((ncov) OR (2019ncov) OR (covid) OR (covid19) OR (sars2) OR (cov 2) OR (cov2) OR (coronavirus*) OR (corona AND virus*) OR (severe AND acute AND respira tory AND syndrome 2) OR (wuhan AND virus) OR (((wuhan) OR (novel) OR (19) OR (2019) OR (epidem*) OR (pandem*) OR (outbreak) OR (new)) AND ((pneum onia AND virus*) OR (cov) OR (hcov)))) OR AFFIL ((wuhan) OR (19) OR (novel) OR (new) OR (2019) OR (epidemy) OR (epidemic*) OR (pandem*) OR (outbreak)) AND TITLE-ABS-KEY ((pneumonia AND virus*) OR (cov) OR (hcov)))

Cochrane library

Concept 1

[mh "Postpartum Period"] OR ("Postpartum" OR "Post-partum" OR "Post-Natal" OR "Postnatal" OR Puerper* OR "after birth" OR "after delivery"):ti,ab,kw

Concept 2

[mh "COVID-19"] OR [mh “SARS-Cov-2”] OR

(nCoV OR 2019nCoV OR COVID OR COVID19 OR SARS2 OR "cov 2" OR cov2 OR coronavirus* OR (corona NEXT virus*) OR "severe acute respiratory syndrome 2" OR “Wuhan virus” OR ((wuhan OR novel OR new OR 19 OR 2019 OR epidem* OR pandem* OR outbreak) AND ([mh ^“coronavirus”] OR [mh ^” Betacoronavirus“] OR [mh ^"Coronavirus Infections"] OR (pneumonia NEXT virus*) OR cov OR hcov))):ti,ab,kw

Concept 3: Depression

[mh "Depressive Disorder"] OR [mh "Depression"] OR [mh "Psychology"] OR [mh "Mental Health"] OR (Psycholog* OR "psychosocial" OR "Mental" OR Depressi* OR "social inability" OR "social ability" OR "emotional" OR "affective"):ti,ab,kw

Concept 4: Postpartum depression

[mh "Depression, Postpartum"]

((1 AND 3) OR 4) AND 2

CINAHL via ebsco

Concept 1

(MH "Postnatal Period+") OR TI ("Postpartum" OR "Post-partum" OR "Post-Natal" OR "Postnatal" OR "Puerper*" OR "after birth" OR "after delivery") OR AB ("Postpartum" OR "Post-partum" OR "Post-Natal" OR "Postnatal" OR "Puerper*" OR "after birth" OR "after delivery")

Concept 2

((MH "COVID-19 Pandemic") OR (MH "COVID-19") OR (MH "SARS-CoV-2") OR

OR TI ("nCoV" OR "2019nCoV" OR "COVID" OR "COVID19" OR "SARS2" OR "cov 2" OR "cov2" OR coronavirus* OR "corona virus*" OR "severe acute respiratory syndrome 2" OR "Wuhan virus")

OR AB ("nCoV" OR "2019nCoV" OR "COVID" OR "COVID19" OR "SARS2" OR "cov 2" OR "cov2" OR coronavirus* OR "corona virus*" OR "severe acute respiratory syndrome 2" OR "Wuhan virus") OR

((TI (wuhan OR novel OR new OR 19 OR 2019 OR epidem* OR pandem* OR outbreak) OR AB (wuhan OR novel OR new OR 19 OR 2019 OR epidem* OR pandem* OR outbreak) OR AF (wuhan OR novel OR new OR 19 OR 2019 OR epidemy OR epidemic* OR pandem* OR outbreak)) AND (TI ("pneumonia virus*" OR "cov" OR "hcov") OR AB ("pneumonia virus*" OR "cov" OR "hcov"))) OR

AF (nCoV OR 2019nCoV OR COVID OR COVID19 OR SARS2 OR "cov 2" OR cov2 OR coronavirus* OR "corona virus*" OR "severe acute respiratory syndrome 2"))

Concept 3: Depression

(MH "Depression+") OR (MH "Psychology+") OR (MH "Mental Health") OR TI (Psycholog* OR "psychosocial" OR "Mental" OR Depressi* OR "social inability" OR "social ability" OR "emotional" OR "affective") OR AB (Psycholog* OR "psychosocial" OR "Mental" OR Depressi* OR "social inability" OR "social ability" OR "emotional" OR "affective")

Concept 4: Postpartum depression

(MH "Depression, Postpartum")

((1 AND 3) OR 4) AND 2
